# When drugs meet disease: disentangling diabetes, obesity, and periconceptional GLP-1 receptor agonist safety

**DOI:** 10.1093/hropen/hoag016

**Published:** 2026-03-18

**Authors:** Yeyi Zhu, Monique M Hedderson

**Affiliations:** Division of Research, Kaiser Permanente Northern California, Pleasanton, CA, USA; Center for Upstream Prevention of Adiposity and Diabetes Mellitus (UPSTREAM), Pleasanton, CA, USA; Division of Research, Kaiser Permanente Northern California, Pleasanton, CA, USA; Center for Upstream Prevention of Adiposity and Diabetes Mellitus (UPSTREAM), Pleasanton, CA, USA

The global surge in the use of glucagon-like peptide-1 receptor agonists (GLP-1 RAs) for both type 2 diabetes and obesity management has fundamentally reshaped cardiometabolic care. As these medications increasingly reach women of reproductive age, the frequency of inadvertent periconceptional exposure is rising. This trend persists despite current manufacturer recommendations to discontinue treatment at least 2 months prior to conception because of their long half-lives and the lack of safety data on their use during pregnancy. Together, these factors have heightened concern about potential reproductive risks while leaving clinicians with limited evidence to guide patient-centered counseling. In this issue of *Human Reproduction Open*, Hviid and colleagues address this timely and critical evidence gap through a nationwide Danish cohort study that examines whether periconceptional GLP-1 RA exposure is associated with adverse obstetric outcomes (Hviid *et al.*, 2026).

Among 756 636 singleton pregnancies between 2009 and 2023, Hviid *et al.* (2026) identified 529 pregnancies with periconceptional GLP-1 RA exposure (295 exposed to liraglutide and 234 to semaglutide) within 8 weeks before or after the last menstrual period. In crude models, the authors initially observed higher rates of several obstetric complications in exposed pregnancies. However, after rigorous propensity score matching that accounted for maternal sociodemographic factors, pre-pregnancy body mass index (BMI), and pre-existing diabetes, periconceptional GLP-1 RA exposure was associated with a higher risk of preterm delivery (PTD), but not with other obstetric complications, only among women with pre-existing diabetes. Specifically, adjusted odds ratios (aOR) for PTD were 1.70 [95% CI, 1.17–2.48] for liraglutide (*n* = 101) and 1.84 (95% CI, 1.24–2.71) for semaglutide (*n* = 110) when used for diabetes management. In contrast, no increased risk of PTD was observed among women using GLP-1 RAs for weight management (liraglutide: *n* = 194, aOR 1.01 [95% CI, 0.58–1.76]; semaglutide: *n* = 124, aOR 0.71 [95% CI, 0.30–1.70]). This divergence supports the authors’ interpretation that it is likely diabetes *per se*, rather than the GLP-1 RA exposure, that drives the adverse obstetric outcomes.

A central strength of Hviid *et al.* (2026) is its explicit stratification by indication (i.e. diabetes treatment versus weight management), which facilitates separation of medication effects from disease-related risk. Diabetes is a well-established independent risk factor for PTD (Sibai *et al.*, 2000). Nonetheless, it is difficult to fully eliminate residual confounding by diabetes severity, duration, or glycemic management, which were not assessed in this study. In this context, residual confounding would likely inflate the observed associations among women treated for diabetes. Accordingly, the divergence in risk profiles between the diabetes and weight-management indications is particularly informative, even if not definitively causal, given the nature of an observational study.

Hviid *et al.* (2026) further conducted a series of sensitivity analyses using counterfactual scenarios to estimate what the risk of obstetric complications would have been if GLP-1 RA-exposed women had not experienced drug-induced weight reduction. Interestingly, the risk estimates for PTD and other complications associated with periconceptional GLP-1 RA exposure remained largely unchanged in these counterfactual models for both the diabetes and weight-management indication groups.

Given that pre-pregnancy obesity is a well-recognized risk factor for perinatal complications, clinical guidelines from organizations such as the American College of Obstetricians and Gynecologists and the International Federation of Gynecology and Obstetrics emphasize that weight management prior to pregnancy is a cornerstone of preconception care to improve obstetric outcomes among individuals with obesity (Hanson *et al.*, 2015; Sagi-Dain 2021). For the diabetes indication group in Hviid *et al.* (2026), the findings in the counterfactual model suggest that the observed risk of PTD was not significantly mitigated by the lower BMI achieved through GLP-1 RAs use. It is plausible that a relatively short-term reduction in periconceptional BMI is insufficient to offset the impact of diabetes-related factors (e.g. hyperglycemia levels, glycemic management) or underlying physiological changes (inflammation, oxidative stress, vascular dysfunction) during the narrow periconceptional window on risk of PTD (Domingueti *et al.*, 2016). For the weight-management indication group in Hviid *et al.* (2026), the null associations in the counterfactual sensitivity analysis provide further reassurance, suggesting that neither the pharmacological exposure nor the associated weight loss appears to compromise obstetric safety in the absence of diabetes.

The findings from this Danish cohort (Hviid *et al.*, 2026) complement and extend an emerging evidence base on the reproductive safety of GLP-1 RAs. Multinational registry and claims analyses spanning four Nordic countries, the USA, and Israel have not shown a higher risk of major congenital malformations with periconceptional GLP-1 RA exposure compared with insulin among pregnancies affected by type 2 diabetes (Cesta *et al.*, 2024). On the other hand, the obstetric-outcome literature remains understudied. In a single-system US study examining GLP-1 RA use with subsequent prepregnancy (within 90 days before pregnancy) or first-trimester discontinuation, exposure was associated with greater gestational weight gain and higher risks of several adverse outcomes, including PTD (Maya *et al.*, 2025). Conversely, a TriNetX-based analysis using a broader preconception window (GLP-1 RA prescriptions within 24 months before pregnancy) reported lower odds of adverse obstetric outcomes, including PTD, consistent with the hypothesis that preconceptional metabolic optimization may confer sustained obstetric benefits (Imbroane *et al.*, 2025). These heterogeneous findings likely reflect differences in exposure definitions and windows, underlying cardiometabolic risk profiles, and the extent to which medication indication can be disentangled from disease. Within this context, Hviid *et al.* (2026) make a distinctive contribution by isolating inadvertent periconceptional exposure, explicitly stratifying by treatment indication, and demonstrating that after rigorous matching, the association with PTD was confined to women treated for diabetes.

As with all observational studies of medication safety in pregnancy, several limitations in the Hviid *et al.* (2026) study merit consideration. Prescriptions do not guarantee ingestion, and registry data often lack detailed information on medication dose, duration of use, and adherence after a prescription is dispensed. Sample sizes, particularly among women using GLP-1 RAs for weight management (an indication approved only recently in many countries) remain modest, limiting power to detect small effects. Furthermore, none of the large-scale registry studies to date have been able to fully adjust for glycemic management, diabetes duration, and other markers of disease severity, or preconception weight trajectory, all of which are known independent risk factors for adverse obstetric outcomes in diabetes-complicated individuals. Nevertheless, Hviid *et al.* (2026) population-based design, comprehensive capture of prescriptions and outcomes, and consistent findings across sensitivity analyses strengthen confidence in the overall conclusions.

For clinicians, the study by Hviid *et al.* (2026) may inform preconception and early pregnancy counseling. The observation that elevated preterm birth risk was confined to women treated for diabetes, and not to those using GLP-1 receptor agonists for weight management, supports more balanced, individualized discussions with patients who may have experienced inadvertent periconceptional exposure. For patients with diabetes, the study reinforces the importance of recognizing diabetes as an important risk factor for obstetric complications and prioritizing metabolic health and glycemic management before and during pregnancy.

For researchers, the Hviid *et al.* (2026) study highlights the enduring challenge of confounding by indication in reproductive pharmacoepidemiology. Indeed, when drugs meet disease, interpretation matters. Future work would benefit from richer clinical data on diabetes duration and severity, treatment history, glycemic management, and weight trajectories, as well as prospective registries capturing dose, medication adherence, discontinuation timing, and early pregnancy losses. As GLP-1 RA use continues to expand, particularly for weight management, coordinated international surveillance across diverse healthcare settings will be essential to refine risk estimates and guide evidence-based counseling.
